# Harnessing cervical lymphatic mechanics to enhance amyloid clearance: a paradigm shift in Alzheimer’s therapeutics?

**DOI:** 10.1186/s40035-025-00521-5

**Published:** 2025-11-06

**Authors:** Wei Wang, Junhui Lv, Zihao Xu, Shuxu Yang, Jin Wang, Weidong Le

**Affiliations:** 1 Department of Neurology, Sir Run Run Shaw Hospital, School of Medicine, Zhejiang University, Hangzhou, China; 2 Department of Neurosurgery, Sir Run Run Shaw Hospital, School of Medicine, Zhejiang University, Hangzhou, China

The ability of the central nervous system (CNS) to clear wastes via cerebrospinal fluid (CSF) outflow is essential for brain health. The cervical lymphatic system is one of the multiple clearance routes for CSF. CSF drains from the subarachnoid space into the meningeal lymphatic vessels, and subsequently into both superficial and deep cervical lymph nodes. This drainage is not only essential for eliminating metabolic waste such as amyloid-β (Aβ) and tau proteins, but also contributes to immune surveillance and the maintenance of interstitial fluid balance [1, 2]. Disruption of this pathway—whether due to aging, inflammation, or structural regression of lymphatic vessels—can lead to accumulation of neurotoxic substances and is increasingly associated with neurodegenerative disorders like Alzheimer’s disease (AD) [3]. In a recent compelling study, Jin et al. (2025) have pushed the frontier of this field by demonstrating that non-invasive mechanical stimulation of superficial cervical lymphatics (scLVs) can enhance CSF clearance, especially in aged mice. This is a breakthrough with potentially wide-ranging clinical implications [4] (Fig. 1).

**Fig. 1:**
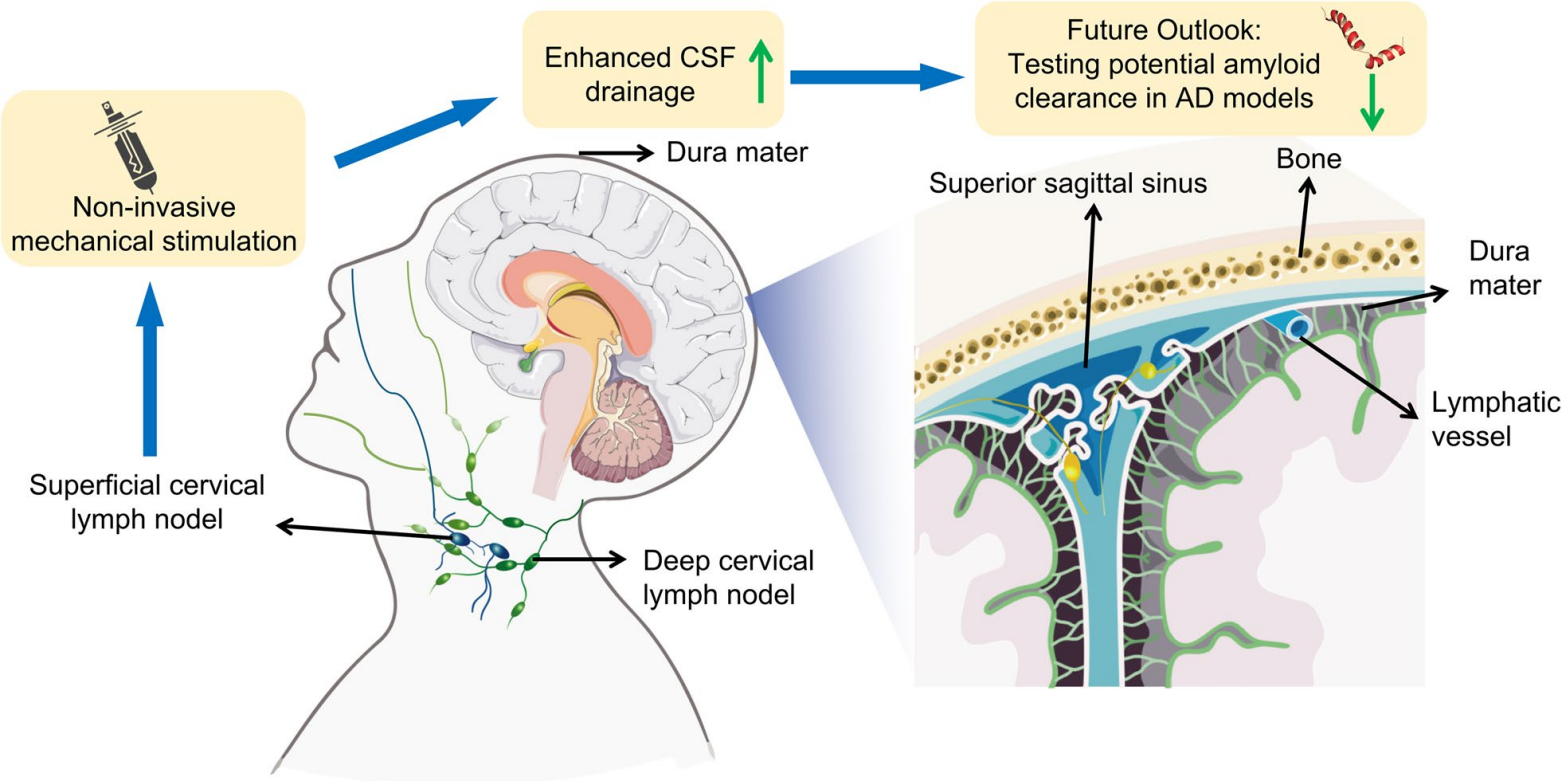
Diagram of increased CSF drainage via non-invasive manipulation of cervical lymphatics. Left, anatomical route of CSF drainage into superficial and deep cervical lymph nodes, highlighting the possibility of external mechanical stimulation. Right, continuity between meningeal lymphatic vessels and the superior sagittal sinus

Access to meningeal and cervical lymphatic pathways, particularly the deep cervical lymphatics, has long been a barrier to intervention. The present work provides an alternative: the scLVs, which are accessible through intact skin [4]. Jin et al. used genetically labeled Prox1–GFP mice and primate models to map the precise anatomical routes of CSF drainage. They revealed that the scLVs drain a substantial amount of the CSF reaching cervical lymph nodes and remain functionally intact in aged animals.

A key innovation of this study is the development of a force-regulated mechanical device that applies low-magnitude, skin-level stimulation along scLV pathways. In aged mice, which have atrophy of upstream lymphatic network, this mechanical intervention increased CSF tracer drainage by up to 4.7 folds, while preserving the natural contractility of the lymphatic vessels. This offers a rare combination of efficacy, safety, and translatability.

This finding addresses an important challenge in neurodegenerative disease management: how to restore impaired CSF clearance in the aged brain. While previous approaches such as vascular endothelial growth factor-C (VEGF-C) delivery, photodynamic therapy, or pharmacologic agents have offered some success, they have potential limitations or systemic side effects. Mechanical stimulation is a non-invasive, localized alternative that could be scaled for clinical use, and has potential for various conditions ranging from AD to traumatic brain injury, where lymphatic drainage is compromised.

Despite the novelty of this technique, several biological caveats constrain its extrapolation to human AD pathogenesis and therapy. While Jin et al. assessed the presence of similar facial lymphatic drainage with a tracer study in non-human primates, they did not demonstrate that facial or neck manipulation produced enhanced drainage in the non-human primate. One key limitation arises from anatomical differences between species. Compared to humans, mice have thinner skulls, less complex dural and meningeal architecture, and relatively more accessible cervical lymphatics. The superficial cervical lymphatic vessels in mice are more superficial and easier to manipulate through transcutaneous mechanical force. In contrast, the lymphatic networks in humans are embedded deeper within soft tissues, and their responses to mechanical stimulation may differ significantly. These structural distinctions may affect both the efficiency and the translation feasibility of such interventions to human subjects.

Second, the failure of ventriculoperitoneal shunt in AD patients despite improved CSF flow suggests that protein clearance is not governed solely by mechanical drainage [5]. Active transendothelial uptake of Aβ by lymphatic endothelial cells—may also be involved in the brain clearance system [6]. However, this pathway is affected by aging. Mechanical stimulation may bypass or partially address this bottleneck. Recent work has further underscored that the development and functional architecture of brain lymphatic systems are under the control of neural activity and glial signaling [7]. Specifically, radial astroglia in the CNS regulate VEGF-C expression in response to neural cues, while meningeal fibroblasts activate this signal via proteolytic maturation, ensuring precise localization of lymphatic endothelial cells and preventing their aberrant invasion into brain parenchyma. This neuro-glia-lymphatic axis suggests that brain lymphatic drainage is not merely passive or mechanical but dynamically sculpted by developmental and activity-dependent signals. Therefore, interventions targeting structural lymphatic flow—such as external mechanical stimulation—must be contextualized within this broader biological framework.

Third, the assumption that cervical lymphatic outflow is critical for cognitive function is challenged by real-world surgical data. For instance, current clinical evidence does not directly show that patients with thyroid cancer who undergo bilateral cervical lymph node dissection (BLND) experience significant long-term cognitive decline. Although numerous studies have shown that patients undergoing BLND surgery have a higher risk of cognitive impairment compared to non-surgical patients, a considerable proportion of individuals did not experience cognitive decline after receiving BLND [8, 9]. This may be due to the compensatory lymphatic rerouting (e.g., via contralateral or deep channels), implying greater plasticity than the model accounts for.

Additionally, the long-term effects of such repeated stimulation on lymphatic endothelial integrity, tissue fibrosis, or inflammatory responses remain to be tested. The current study by Jin et al. did not address whether chronic stimulation over weeks or months may induce maladaptive changes in the lymphatic vessels. Mechanical stress is known to modulate endothelial biology in both blood and lymphatic systems, and persistent activation could theoretically compromise vessel function through inflammation, fibrosis, or structural remodeling [10]. Therefore, thorough long-term safety assessments, including histological and molecular analyses of the stimulated lymphatics, are crucial before any translational application in humans can be considered. While the enhanced drainage of tracers provides preliminary evidence of lymphatic activation, these findings cannot be directly interpreted as the clearance of pathogenic proteins such as Aβ or tau, since the transport properties of tracers are not necessarily equivalent to those of disease-related molecules. Finally, the absence of biochemical, behavioral, and neurophysiological correlates highlights the need for future studies to integrate functional outcomes in order to establish the translational potential of cervical lymphatic manipulation.

In summary, the work by Jin et al. introduces a novel biophysical approach to enhance brain clearance through cervical lymphatic modulation. Nevertheless, several physiological, anatomical, and translational caveats, particularly the active role of lymphatic endothelium, the presence of compensatory pathways, and the nature of lymphatic regression with aging, underscore the need for caution when considering direct clinical applications. Future investigations in AD animal models and patient cohorts will be essential to determine whether cervical lymphatic modulation can indeed facilitate the clearance of pathological proteins and ultimately translate into meaningful therapeutic benefit.

## Data Availability

Not applicable.

## Abbreviations

CNS: Central nervous system
CSF: Cerebrospinal fluid
AD: Alzheimer’s disease
scLVs: Superficial cervical lymphatics
VEGF-C: Vascular endothelial growth factor-C
BLND: Bilateral cervical lymph node dissection
